# Blood-Brain Delivery Methods Using Nanotechnology

**DOI:** 10.3390/pharmaceutics10040269

**Published:** 2018-12-11

**Authors:** Daniel Mihai Teleanu, Cristina Chircov, Alexandru Mihai Grumezescu, Adrian Volceanov, Raluca Ioana Teleanu

**Affiliations:** 1 Emergency University Hospital, Bucharest, Romania, “Carol Davila” University of Medicine and Pharmacy, 050474 Bucharest, Romania; telepapa@hotmail.com (D.M.T.); raluca.teleanu@umfcd.ro (R.I.T.); 2 Faculty of Engineering in Foreign Languages, Politehnica University of Bucharest, 060042 București, Romania; cristina.chircov@yahoo.com; 3 Department of Science and Engineering of Oxide Materials and Nanomaterials, Faculty of Applied Chemistry and Materials Science, Politehnica University of Bucharest, 060042 București, Romania; grumezescu@yahoo.com; 4 ICUB-Research Institute of University of Bucharest, University of Bucharest, 36-46 M. Kogalniceanu Blvd., 050107 Bucharest, Romania

**Keywords:** brain, brain cancer, Alzheimer’s disease, Parkinson’s disease, nanoparticles, brain delivery, liposomes, dendrimers, micelles, carbon nanotubes

## Abstract

Pathologies of the brain, of which brain cancer, Alzheimer’s disease, Parkinson’s disease, stroke, and multiple sclerosis, are some of the most prevalent, and that presently are poorly treated due to the difficulties associated with drug development, administration, and targeting to the brain. The existence of the blood-brain barrier, a selective permeability system which acts as a local gateway against circulating foreign substances, represents the key challenge for the delivery of therapeutic agents to the brain. However, the development of nanotechnology-based approaches for brain delivery, such as nanoparticles, liposomes, dendrimers, micelles, and carbon nanotubes, might be the solution for improved brain therapies.

## 1. Introduction

Brain diseases, including brain cancer, Alzheimer’s disease, Parkinson’s disease, stroke, and multiple sclerosis, are some of the most prevalent diseases, which are becoming a great concern due to the increase in elderly population [1]. These disorders may be caused by genetic and environmental factors, pathologies in processes involving protein aggregation which lead to neurodegeneration or dysregulation of the immune process, or abnormalities regarding the development and function of the brain [2]. However, compared to other areas of an organism, the treatment for brain diseases is presently unsuccessful mostly due to the complexity of the brain. Additionally, drug development for brain diseases requires longer periods of time and more complex clinical trials. Since the number of cases are expected to increase over the following years, the discovery of novel and improved strategies is crucial [3].

The pathways for the delivery of therapeutic agents to the brain can either be invasive or non-invasive. The invasive route involves the surgical administration of drugs directly inside the brain, thus providing a sufficient dosage without causing systemic toxicity. However, the intracerebral injection relies on the cerebral diffusion, thus being concentration-dependent and decreasing from the administration site. The non-invasive administration strategies are based on the anatomical structure of the brain capillaries, cells, and extracellular environment, and on the directional transfer of fluids across the brain, the main routes including the nasal and the systemic administration [4]. The nasal route is preferred over the systemic drug delivery as the drug is directly delivered into the brain through the olfactory bulb, which increases the bioavailability and reduces the degradation of the drug. Nevertheless, limitations such as poor drug permeations through the nasal mucosa and mucociliary clearance might be encountered [5]. Considering the systemic route, the circulating drugs must enter the parenchyma and the cerebrospinal fluid and further diffuse through the brain extracellular space to the targeted site [4].

The brain is one of the most complex and important organs of living organisms. Therefore, it is necessary to protect it against the contamination with environmental and foreign substances which could lead to changes in the inner and outer concentrations of neuronal cells and subsequently to impairments in nerve conduction and dysfunctions in the body control processes [6]. The blood-brain barrier is the structure responsible for the protection of the brain, acting as a local gateway against the circulating toxins and cells [7] through a selective permeability system. Hence, the delivery systems for the treatment of brain diseases should have the capacity to cross the blood-brain barrier without causing immune responses. However, the physiological function of the blood-brain barrier is the key challenge for the delivery of pharmaceutical drugs to the brain, which represents the main reason for complications in the existing treatment strategies and for the numerous research studies focusing on the development of novel drug delivery systems for the treatment of brain-associated diseases [8]. The principal pathways for crossing the blood-brain barrier are through paracellular transport, between endothelial cells, and through transcellular transport, involving passive or active mechanisms, across the luminal side of the endothelial cells, through the cytoplasm, and subsequently across the abluminal side, into the brain interstitium [9]. Although there are multiple crossing pathways, approximately 98% of small molecules and most large molecules are unable to reach the brain through the blood-brain barrier [10].

Nanotechnology, the emerging field that encompasses knowledge from multiple disciplines including chemistry, physics, engineering, and biology, implicates the development and modification of materials within the size range of 1–100 nm in at least one dimension [11,12,13]. Additionally, nanotechnology represents the capacity to understand, manipulate, and control the matter at the level of individual atoms and molecules [14]. Therefore, the implication of nanotechnology for the development of non-invasive drug delivery strategies could lead to the design of novel and improved formulations to enhance the delivery of therapeutic agents across the blood-brain barrier [3,15,16,17,18]. Numerous research studies have focused on the exploration of nanotechnology-based drug delivery systems, including nanoparticles, liposomes, dendrimers, carbon nanotubes, and micelles, which have the potential to deliver the desired quantity of the drug to the brain [10].

## 2. The Blood-Brain Barrier

The central nervous system compartment, consisting of the brain and the spinal cord, is protected by two main barriers: the blood-brain barrier, formed by the brain microvascular endothelial cells, and the blood-cerebrospinal fluid barrier, comprised of the epithelial layer of choroid plexus, the cerebral ventricles, and the arachnoid mater covering the outer brain surface [19]. As the subject of this review, the blood-brain barrier is characterized by its unique structure and the highly controlled interactions between its cellular and acellular components. The main function of the blood-brain barrier is to ensure an optimal environment for the proper functionality of the neuronal network, by maintaining brain homeostasis, regulating the influx and efflux of fluids, and protecting the brain against pathogenic agents [20] through a dynamic combination of cellular, vascular, molecular, and ionic factors. Additionally, it contributes to the neuronal functionality by allowing the glucose transport [21].

### 2.1. The Anatomical Structure of the Blood-Brain Barrier

**Figure 1:**
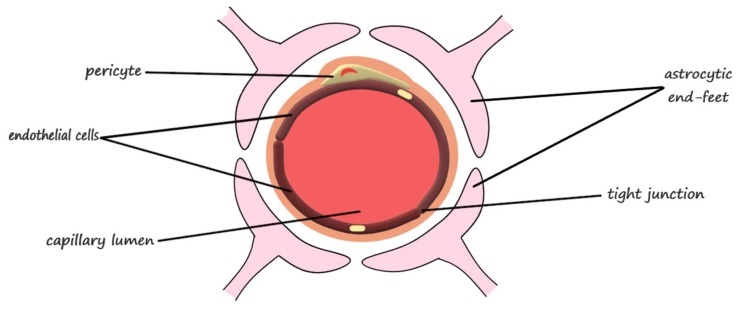
The main structural components of the blood-brain barrier.

The main component of the blood-brain barrier is the continuous layer of endothelial cells connected through tight junctions composed of claudin-5, occludin, and other molecules [22], which represents the luminal surface of more than 99% of the capillaries of the brain and spinal cord [23]. Additionally, the blood-brain barrier is composed of specialized cells, including the pericytes, the astrocytes, and the adjacent neurons (Figure 1) [24]. The functions associated to the main blood-brain barrier components are summarized in Table 1.

The endothelium of the brain provides a surface area of 20 m^2^ for the blood-brain exchange, while the tight junctions direct the molecular trafficking across the blood-brain barrier through the transcellular route [25]. The influx of necessary substances and the efflux of waste are enabled by the structural and functional unit of the central nervous system, which is the neurovascular unit [24]. This structure is formed at the contact sites between small blood vessels, such as arterioles, precapillary arterioles, capillaries, and postcapillary venules, and the parenchymal cells of the brain. The wall of the blood vessels consists of three different types of layers. The inner layer of endothelial cells is separated from the middle layer of contractile cells through the basement membrane. Subsequently, the outer layer differs depending on the size of the vessel: in arteries and arterioles it consists of smooth muscle cells, in capillaries, the smooth muscle cells are replaced by pericytes, and larger vessels are surrounded by an adventitial layer, which contains perivascular nerve endings of extracerebral origin for the pial vasculature and intrinsic nerves originating from subcortical areas or local interneurons for the intraparenchymal vasculature, and are separated from the parenchyma by the Virchow-Robin space [26].

The paracellular space between endothelial cells is eliminated through the tight junctions, which appear as continuous, anastomosing, intramembranous networks of strands and interact with the tight junction proteins on the adjacent endothelial cells. Therefore, the transfer of solutes and ions between the brain and the blood is blocked by the fusion of tight junctions [27].

Pericytes, spatially isolated contractile cells found in the perivascular space, are involved in angiogenesis, maintenance of the blood-brain barrier, regulation of immune cell entry to the brain, control of the cerebral blood flow, and constriction of capillaries in stroke [28]. Astrocytes, also known as astroglia, the most abundant cells of the human brain [29], are responsible for the regulation of metabolism, the modulation of neuronal transmission, and brain development and repair. The astrocytic end-feet processes that surround the endothelial cells, over the basal lamina, termed glia limitans, forms a highly restrictive second barrier [22]. Neurons are actively involved through the nervous terminations that reach all the cells that form the blood-brain barrier [30].

### 2.2. The Physiology of the Blood-Brain Barrier

Numerous research works have focused on the study of strategies to safely and efficiently cross the brain capillary endothelium by therapeutics and drug delivery systems. There are multiple ways for small molecules and biomacromolecules to cross the blood-brain barrier, primarily either through paracellular or transcellular pathways [32].

**Figure 2:**
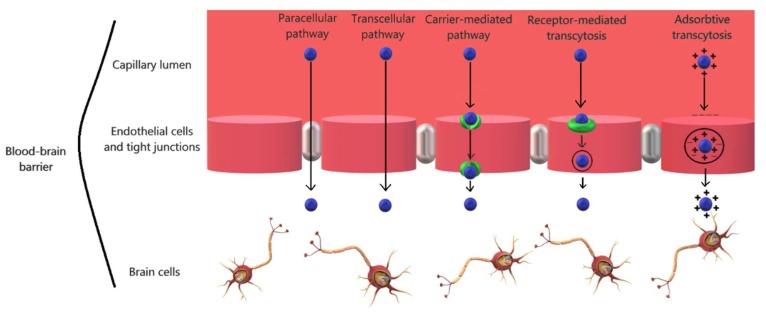
Nano-carrier delivery pathways across the blood-brain barrier.

For small molecules, there are three main routes to cross the blood-brain barrier and reach the brain parenchyma under physiological conditions (Figure 2), depending on their characteristics [33]. Firstly, the paracellular transportation through an aqueous pathway is specific for small hydrophilic molecules, but it is limited by the regulation of the transient relaxation of tight junctions between the endothelial cells [32]. Secondly, it has been demonstrated that small lipophilic molecules enter the brain tissue through transcellular diffusion, a non-saturable mechanism. However, the route of transcellular diffusion involves the traversing of the luminal membrane, cytosol, and subsequently the abluminal membrane prior to reaching the brain tissue, which represents a challenge due to the tendency of lipophilic substances to be captured within the cell membrane. Furthermore, the diffusion might also be affected if the substance is an efflux transporter substrate [34]. Thirdly, most of the remaining small molecules reach the brain through a substrate-specific process, the endogenous transporter- or carrier-mediated pathway, which is driven by the concentration gradient of the substrates with the assistance of suitable transporters [33].

Regarding the large biological molecules, such as peptides and proteins, the mechanisms for crossing the blood-brain barrier are through endocytosis. There are seven main specific and non-specific mechanisms of penetration, specifically the passive pathways across the tight junctions or through the transcellular lipophilic route and the active pathways, through transcytosis, the leading approach which consists of active efflux carriers transcytosis, carrier-mediated transcytosis, receptor-mediated transcytosis, and adsorptive mediated transcytosis (Figure 2), and through tight junction modulation [35].

In addition to structure modification of drug molecules strategies, such as increasing the lipophilicity or reducing the molecular size, the blood-brain barrier permeation can be improved by focusing on reducing the efflux transport, enhancing the transcellular diffusion permeability [36], or by transiently disrupting the tight junction complexes, which are obtained through technologies such as targeted suppression of tight junction protein expression by using RNA interference. Furthermore, nanomedicines and molecular Trojan horses represent novel and emerging approaches for the delivery of drug molecules across the blood-brain barrier [37].

## 3. Nanotechnology Approaches for Crossing the Blood-Brain Barrier

The development of nanotechnology through integrated multidisciplinary efforts will result in novel insights into the functions of neural circuits and approaches for the diagnostic and therapy of brain diseases [38]. This is especially necessary due to the limitations of current strategies to deliver drugs into the central nervous system through the blood-brain barrier. The specific properties of nanomaterials, such as reduced size, biocompatibility, prolonged blood circulation, and non-toxicity, have been exploited for the creation of an emerging delivery platform that can easily transport therapeutic agents to the brain [39]. The nanotechnology-mediated drug delivery systems are based on both specific and non-specific mechanisms for targeting brain sites [40]. Recent studies have focused on the development of drug delivery nano-vehicles, including nanoparticles, liposomes, dendrimers, micelles, and carbon nanotubes, for the delivery of pharmaceutical agents, peptides, proteins, vaccines, or nucleic acids. The parameters of the main nano-carriers used for drug delivery across the blood-brain barrier are summarized in Table 2.

### 3.1. Organic Nanomaterials

#### 3.1.1. Polymeric Nanoparticles

As nanoparticles possess suitable properties for drug delivery, such as controlled drug release and targeting efficiency, they have been widely used for the development of drug delivery carriers to cross the blood-brain barrier. Moreover, they can avoid phagocytosis by the reticuloendothelial system, thus improving the concentration of drugs in the brain [41]. Several types of nanoparticles have been studied for the efficient blood-brain barrier crossing, including polymeric and inorganic nanoparticles.

Recent studies have focused on the use of poly(lactide-*co*-glycolic) acid as a material for the synthesis of nanoparticles to encapsulate therapeutic agents for the treatment of Alzheimer’s disease [42] and brain cancer [43,44]. In vitro studies showed that the use of polymeric nanoparticles enhanced drug delivery to the brain, with reduced oxidative stress, inflammation and plaque load through the improved delivery of curcumin for treating Alzheimer’s disease [42], and efficient internalization of doxorubicin into the human glioma cells, resulting in cytotoxic effect on cancer cells [43]. Additionally, the in vivo experiment regarding the co-delivery of cisplatin and boldine, an antioxidant agent, using the poly(lactide-*co*-glycolic) nanocarriers resulted in an effective target-specific delivery for therapeutic use in brain cancer therapy [44]. Furthermore, the use of a positively charged polymer, poly(ethylene imine) [45], and of the poly(ethylene imine)-poly(l-lysine) copolymer [46] as gene delivery vehicles has been reported. To improve the cytocompatibility of poly(ethylene imine), l-glutathione was attached to the backbone of the polymer, which also enhanced the passage through the blood-brain barrier in vitro [45]. Thus, it has been demonstrated the potential of poly(ethylene imine)-based nanoparticles for the delivery of genes for gene therapy in brain cancer [45,46]. Another polymer used for the synthesis of nanoparticles for brain delivery is poly(allylamine) hydrochloride. The encapsulation of kynurenic acid into the core-shell structures has indicated neuroprotective properties and therapeutic potential for neurological disorders, in both in vitro and in vivo experiments [47]. Other studies have focused on the use of andrographolide loaded into human serum albumin-based nanoparticles and polyethylcyanoacrylate nanoparticles for the treatment of inflammation related to neurodegenerative diseases. Results showed a slightly increased permeability for the human serum albumin nanoparticles, while the polyethylcyanoacrylate nanoparticles reversibly disrupted the integrity of the cell monolayer utilized for the in vitro experiment [61]. The delivery of docetaxel for the treatment of brain metastasis has been achieved through the development of penetrating amphiphilic polymer-lipid nanoparticles system. The in vivo tests indicated the accumulation of the nanoparticles at the tumor site, with effectively inhibited tumor growth, and increased median survival compared to an equivalent dose of clinically used docetaxel solution formulation [48]. Chitosan conjugated with l-valine has been utilized as vehicle to deliver saxagliptin, a hydrophilic therapeutic agent, for the therapy of Alzheimer’s disease. In the in vivo studies, the nanoparticles showed plasma stability, thus preventing premature release, and enhanced brain delivery compared to the suspension of saxagliptin [62].

#### 3.1.2. Liposomes

Liposomes are synthetic and spherical cells, consisting of single amphiphilic lipid bilayers, which can entrap therapeutic molecules, including drugs, vaccines, nucleic acids, and proteins. Thus, they have been extensively used as drug delivery systems to enhance the safety and effectiveness of the therapeutics [63].

The applications of liposomes mostly target brain cancer therapy, due to the capacity to cross the blood-brain barrier and deliver an appropriate quantity of drugs to the brain. Many studies have reported the use of liposomal formulations to deliver anti-cancer drugs, such as methotrexate [64], 5-fluorouracil [65], paclitaxel [66], doxorubicin [49,50], and erlotinib [49]. In order to improve the efficiency of the blood-brain barrier passage of the liposomes, there are a few strategies that may be applied to the formulations. Thus, liposomes can be coated with various molecules: poly(ethylene glycol) has shown to extend the blood–circulation time of the formulations [64], transferrin for receptor targeting lead to enhanced translocation of the carriers across the brain [49,65], and the glucose-vitamin C complex has improved the accumulation of liposomes at the targeted site [66]. Additionally, in silico experiments demonstrated the capacity to facilitate a favorable hydraulic environment around the infusion site to enhance drug transport locally through convection enhanced delivery of liposomes [50].

Furthermore, the inhibition of β-amyloid-induced Alzheimer’s disease using a drug carrier system of apolipoprotein E-modified liposomes conjugated with phosphatidic acid designed to improve blood-brain barrier penetration and release quercetin and rosmarinic acid has been reported [51]. Another treatment strategy involves the use of transferrin modified liposomes to deliver α-mangostin, a potential candidate for neurodegenerative diseases therapy [67]. Both in vitro and in vivo studies showed enhanced blood-brain barrier permeation and efficient delivery of drugs.

Liposomes have also been utilized as carriers for gene therapy. Research works have reported the delivery of oligonucleotides for brain cancer therapy using mannitol for blood-brain barrier disruption [68] and the delivery of liposomes functionalized with transferrin receptor targeting and penetratin for enhanced cell penetration for the efficient delivery of nucleic acids for brain diseases treatment [52].

#### 3.1.3. Dendrimers

Dendrimers are a class of synthetic macromolecules, with a tree-like topology, characterized by defined molecular weights and specific encapsulation properties. The globular structure of dendrimers comprises a core, layers of branched repeat units emerging from the core, and functional end groups on the outside layer [69].

As nanotechnology-based strategies to overcome the blood-brain barrier, dendrimers have been applied for the treatment of brain cancer, neurodegenerative diseases, stroke, neuroinflammation, and circulatory arrest. Poly(ethylene glycol) and glioma homing peptides conjugated polyamidoamine dendrimers were synthesized for targeting glioblastomas, in vivo results indicating enhanced tumor penetration [70]. Moreover, sialic acid, glucosamine and concanavalin A anchored poly(propyleneimine) dendritic nanoconjugates used to deliver paclitaxel significantly increase the amount of drug at the tumor site, with a reduced efflux of the nanocarriers system [71].

Polyamidoamine dendrimers are the most commonly studied dendrimers for brain diseases treatment. The encapsulation of carbamazepine, an anti-epileptic drug, has been reported for the treatment of Alzheimer’s disease [53]. Poly(ethylene glycol) conjugated polyamidoamine dendrimers have also been used as vehicles for the delivery of drugs and reducing blood clotting for ischemic stroke therapy [54]. The delivery of drugs across the blood-brain barrier to treat hypothermic circulatory arrest was achieved by using generation 6 dendrimers, with higher dendrimer accumulation correlated to higher levels of proinflammatory cytokines and specific targeting of microglia and injured neuronal cells [72]. The inhibition of neuroinflammation mechanisms has been studied for cerebral palsy treatment, with dendrimer uptake directly correlated to the severity of the disease [73]. Furthermore, the presence of penetration enhancer capmul increases the absorption to the brain, thus proving the potential for developing an oral *N*-acetyl-l-cysteine conjugated dendrimer formulation for the treatment of neuroinflammation [74].

#### 3.1.4. Micelles

Micelles are amphiphilic molecules, with a particle size within the range of 5–50 nm. Micelles form spontaneously under specific conditions of concentration and temperature of the aqueous solution [75]. The mechanism involves the self-assembly of amphiphilic molecules, with the hydrophilic/polar region, known as the head, facing the outside surface and the hydrophobic/non-polar region, known as the tail, forming the core. Micelles have attracted the attention for the delivery of poorly water-soluble molecules, providing sustained and controlled release, chemical and physical stability of the drugs, and improving drug bioavailability [76].

The potential of polymeric micelles has been evaluated for improving the blood-brain barrier passage of drugs for brain diseases therapy. Several block copolymers have been studied, including poly(styrene)-poly(acrylic acid), poly(ethylene glycol)-*b*-poly(lactic acid), and distearyl-sn-glycero-3-phosphoethanolamine-*N*-methoxy poly(ethylene glycol), of which the latter provided enhanced cellular uptake, proving the potential of polymeric micelles to deliver encapsulated drugs to the specific brain sites [77]. Furthermore, the development of targeting and cellular uptake mediating lipopeptide derived from apolipoprotein E, that forms micelles and rapidly incorporates into liposomes have led to the conclusion that the size of the carriers and the surface density of cationic peptides are key determinants for the development of target specific drug delivery systems to the brain [78]. Recent works have focused on the delivery of curcumin for targeting glioma [79] and treating Alzheimer’s disease [55] using micelles as nanocarriers. Additionally, micelles carrying contrast agents might be applied for the magnetic resonance imaging of neuroinflammation [56] and ischemic stroke injuries [57].

### 3.2. Inorganic Nanomaterials

Inorganic nanomaterials have been widely used in biomedical applications. However, since they lack the property of biodegradability, most applications aim the tissue bioimaging for diagnostics. Nonetheless, several inorganic nanoparticles, including gold and silica nanoparticles, and carbon nanotubes have been used to deliver specific drugs across the blood-brain barrier.

#### 3.2.1. Gold Nanoparticles

Gold nanoparticles have been commonly studied in the therapy of neurodegenerative diseases through the functionalization with therapeutic macromolecules. The treatment of Alzheimer’s disease by using gold nanoparticles functionalized with β-amyloid specific peptides [80] and the treatment of Parkinson’s disease with l-DOPA functionalized multi-branched nanoflower-like gold nanoparticles [81] have been studied, showing enhanced blood-brain barrier permeability across in vitro models. Furthermore, the effect of insulin-coated gold nanoparticle size on the capacity to overcome the blood-brain barrier has been studied. Results showed that the smallest nanoparticles, with the diameter of 20 nm presented the most widespread biodistribution and accumulation within the brain [82]. Similarly, insulin-coated gold nanoparticles with 20 nm in diameter have been injected into the tail vein of mice. Microcomputed tomography images showed an accumulation in the mice brains, demonstrating the effectiveness of these nanosystems in imaging diagnosis of brain diseases and in the delivery of therapeutic agents [83]. Moreover, transactivator of transcription peptide-modified gold nanoparticles with a 5-nm core size containing doxorubicin, an anticancer drug, and gadolinium chelates as imaging contrast agents have been administered for theranostic applications in glioblastoma. In vitro and in vivo results proved the potential of these nano-carriers to penetrate the blood-brain barrier and efficiently deliver anticancer drugs and enhance brain tumor imaging [84].

Another research work focused on the use of citrate and polyethylene glycol-coated gold nanoparticles for visualizing cortical vasculature changes, which are associated with disruptions in the blood-brain barrier. Nanoparticles were administered to mouse models of stroke and the multi-photon luminescence imaging proved the capacity of the nanoplatforms for monitoring vascular morphology and physiology associated with brain diseases. Furthermore, nanoparticles with 5 nm or smaller diameters could be useful for the diagnostics of early stages of blood-brain barrier dysfunctions and for drug delivery [85].

#### 3.2.2. Silica Nanoparticles

Surface modified fluorescent silica nanoparticle derivatives have shown potential for applying them as nano-vehicles for drug delivery to the brain considering the possibility of attaching various types of molecules to the core [86]. By attaching lactoferrin on the surface of the polyethylene glycol-coated silica nanoparticles, the process of receptor-mediated transcytosis of these nanosystems has been enhanced, with a maximum transport efficiency observed for nanoparticles with 25 nm in diameter. Thus, these nanosystems could be employed for drugs and imaging probes delivery across the blood-brain barrier [87]. The application of silica nanoparticles to deliver nootropics, such as piracetam, pentoxifylline, and pyridoxine, that are designed to enhance the permeability of the blood-brain barrier has been reported. The efficiency of silica nanoparticles as nanocarriers for drugs in comparison to the unencapsulated drugs has been demonstrated, since the latter were not detected in the brains of the mice [58].

A comparative study between spherical and rod-shaped bare mesoporous silica nanoparticles and poly(ethylene glycol)-poly(ethylene imine)-coated mesoporous silica nanoparticles has been conducted. Although the effect of the shape on the blood-brain barrier permeability, coating the nanoparticles greatly enhanced the cellular uptake for the in vitro models. However, the in vivo imaging experiments on mice showed no blood-brain barrier penetration, which could be associated to the large dimensions of the particles, ranging from 50 to 240 nm [88]. Another study on zebrafish embryos demonstrated that blood-brain barrier penetration is dependent on the surface charge and the size of the nanoparticles, with enhanced transport capacity related to negative charges and reduced sizes [Chen C-T, Chen Y-P, Wu S-H, Chang T-Y, Chou C-M. Negatively charged mesoporous silica nanoparticles penetrate through the Zebrafish larval blood-brain barrier. EuroSciCon Conference on Nanotech and Nanobiotechnology Nano; Paris. Nano Research and Applications; 2018.] Polylactic acid-coated mesoporous silica nanoparticles conjugated with a ligand peptide of low-density lipoprotein receptor to enhance the transcytosis process across the blood-brain barrier have been employed in the delivery of resveratrol, a therapeutic agent for excess reactive oxygen species and reactive nitrogen species removal. The in vitro study showed the potential of the 200 nm nanoparticles in antioxidant-based therapy for neurodegenerative diseases and neural injuries treatment [89].

#### 3.2.3. Carbon Nanotubes

Carbon nanotubes are a class of nanomaterials, consisting of graphite sheets tubes with nano-scaled diameters. Carbon nanotubes can be single-walled or multi-walled, with open ends or closed with fullerene caps [90]. Recently, they have gained a great interest as nanocarrier systems, due to the possibility of functionalization with specific chemical compounds, thus modifying their physical and biological properties. Additionally, carbon nanotubes can be applied cancer therapy through photothermal action [91].

Polymer-coated carbon nanodots [60] and chemically-functionalized multi-walled carbon nanotubes [59] have been applied for the delivery of drugs for brain cancer therapy. Both in vitro and in vivo experiments indicated the penetration of the blood-brain barrier and enhanced uptake in tumors [59,60]. For the treatment of Alzheimer’s disease, berberine, an isoquinoline alkaloid, used for the management of dementia and other neurological disorders, has been adsorbed onto the surface of multi-walled carbon nanotubes. Comparing to the administration of the pure drug, this drug delivery system significantly improved drug absorption in the brain, with potential in reducing β-amyloid induced Alzheimer’s disease [92].

The permeation of amino-functionalized multi-walled carbon nanotubes through the blood-brain barrier has been studied in vitro, using a co-culture model comprising primary porcine brain endothelial cells and primary rat astrocytes, and in vivo, through the systemic administration in mice. The results of the study could pave the way for carbon nanotubes application in the delivery of drugs and biologics to the brain, causing no toxic effects on the cells [93].

## 4. Conclusions and Perspectives

Drug targeting and delivery to the brain represent key challenges due to presence of the blood-brain barrier, which is responsible for the protection of the brain against foreign substances. In order to progress in the effective treatment of brain cancer, neurodegenerative disease, and stroke, which are highly prevalent diseases, novel strategies for the enhanced passage of the blood-brain barrier must be developed. Nanotechnology-based approaches are intensively studied at the moment, including nanoparticles, liposomes, dendrimers, micelles, and carbon nanotubes as nanocarriers to overcome the blood-brain barrier and deliver the appropriate amount of drug to the specific brain site. Further research is needed to understand and mediate the blood-brain barrier crossing mechanisms and to improve the efficiency of brain delivery methods using nanotechnology.

## Figures and Tables

**Table 1:** The main components of the blood-brain barrier and the associated functions.

	Blood-Brain Barrier Component	Function
Neurovascular unit	endothelial cells	barrier function, transport of micronutrients and macronutrients, receptor-mediated signaling, leukocyte trafficking, and osmoregulation [31]
	astrocytes	responsible for proper neuron and neurovascular unit functions and modulation of the blood-brain barrier phenotype [31] regulation of metabolism, the modulation of neuronal transmission, and brain development and repair [22]
	pericytes	regulation of endothelial cell proliferation, survival, migration, differentiation, and vascular branching [31] involved in angiogenesis, maintenance of the blood-brain barrier, regulation of immune cell entry to the brain, control of the cerebral blood flow, and constriction of capillaries in stroke [28]
	neurons	modulation of the blood-brain barrier permeability through neuronal-microvascular communications [31]
	extracellular matrix	modulation of the blood-brain barrier permeability and maintenance of tight junctions [31]
Junctional complexes	occludin	ensures a high electrical resistance (tightness) of the tight junctions [31]
	claudins	primary barrier function of the tight junctions [31]
	junctional adhesion molecules	mediation of the early attachment of adjacent cell membranes, involved in developmental processes [31]
	membrane-associated guanylate kinase-like proteins	modulation of the blood-brain barrier permeability [31]

**Table 2:** The parameters (diameter, surface charge, and cellular uptake) of the nano-carriers reviewed in this paper.

Nano-Carrier			Diameter (nm)	Surface Charge (mV)	Cellular Uptake (%)
Organic nanomaterials	Polymeric nanoparticles	poly(lactide-*co*-glycolic) acid	200–250	(−22)–(−13)	75 [41]
			120 114 143	−11.6 −14.9 −30.8	n.r. 90 90 [42]
			115	−17.4	17.46 [43,44]
		poly(ethylene imine)	104–160 277–287 116–118	28.4 −6.9 33.2	~100 <10 ~100 [43,44]
		poly(ethylene imine)-poly(l-lysine) copolymer	136	30	n.r. [45]
		poly(allylamine) hydrochloride	106.5–113.5	n.r.	n.r. [46]
		human serum albumin	221.9–228.3	−12.3	n.r. [47]
		polyethylcyanoacrylate	218–241.1	(−3.85)–(−2.78)	n.r. [47]
		chitosan	300–324	0.584	n.r. [48]
	Liposomes		173.45–182.79	0.56−3.68	60–70 [49]
			105–110	(−5)–(−2)	70–90 [50]
			158.7–165.05	7.66	65–70 [51]
			189.21–203.39	−22.23	n.r. [52]
	Dendrimers	polyamidoamine	5.1–8.2	2.07–3.15	n.r. [53]
		poly(propyleneimine)	37.8–47.6	18.2	n.r. [54]
	Micelles		11.7–24.9	−30–20	n.r. [55]
			74.2	−30.25	n.r. [56]
			28.79	−6.46	n.r. [57]
Inorganic nanomaterials	Gold nanoparticles		1.4–60.2	−64–56	n.r. [50]
	Silica nanoparticles		120–128	n.r.	n.r. [58]
	Carbon nanotubes		125–296	n.r.	n.r. [59,60]

n.r., not reported in the article as percentage of cellular uptake.
